# mRNA Vaccines: The Dawn of a New Era of Cancer Immunotherapy

**DOI:** 10.3389/fimmu.2022.887125

**Published:** 2022-06-02

**Authors:** Zhuoya Deng, Yuying Tian, Jianxun Song, Guangwen An, Penghui Yang

**Affiliations:** ^1^Department of Infectious Diseases, The Fifth Medical Center of PLA General Hospital, Beijing, China; ^2^Department of Microbial Pathogenesis and Immunology, College of Medicine, Texas A&M University, Bryan, TX, United States; ^3^Department of Pharmacy, No. 984 Hospital of the PLA, Beijing, China

**Keywords:** mRNA vaccine, malignant tumor, delivery system, immunological mechanism, clinical trials

## Abstract

mRNA therapy is a novel anticancer strategy based on *in vitro* transcription (IVT), which has potential for the treatment of malignant tumors. The outbreak of the COVID-19 pandemic in the early 21st century has promoted the application of mRNA technologies in SARS-CoV-2 vaccines, and there has been a great deal of interest in the research and development of mRNA cancer vaccines. There has been progress in a number of key technologies, including mRNA production strategies, delivery systems, antitumor immune strategies, etc. These technologies have accelerated the progress and clinical applications of mRNA therapy, overcoming problems encountered in the past, such as instability, inefficient delivery, and weak immunogenicity of mRNA vaccines. This review provides a detailed overview of the production, delivery systems, immunological mechanisms, and antitumor immune response strategies for mRNA cancer vaccines. We list some mRNA cancer vaccines that are candidates for cancer treatment and discuss clinical trials in the field of tumor immunotherapy. In addition, we discuss the immunological mechanism of action by which mRNA vaccines destroy tumors as well as challenges and prospects for the future.

## 1 Introduction

Despite remarkable progress in oncology, malignant tumors remain the second leading cause of mortality worldwide (1). Conventional clinical treatments for tumors include surgery, radiotherapy, chemotherapy, targeted therapy, immunotherapy, and combination therapy. In addition, the effective treatment of several malignancies with immune checkpoint inhibitors (CPIs) has inspired new ideas about tumor immunotherapy (2). Tumor immunotherapy is aimed at activating the host’s antitumor immunity, leading to a tumor-suppressive microenvironment and, ultimately, achieving tumor shrinkage and improving the overall survival of patients. Cancer vaccines are promising means of antitumor immunotherapy. Vaccines against tumor-associated antigens (TAAs) or tumor-specific antigens (TSAs) can specifically attack and destroy malignant tumor cells with high-level expression of these antigens, and achieve sustained tumor killing through immune memory. Therefore, in comparison to other types of immunotherapy, cancer vaccines could theoretically provide specific, safe, and well-tolerated therapy.

**Figure 1:**
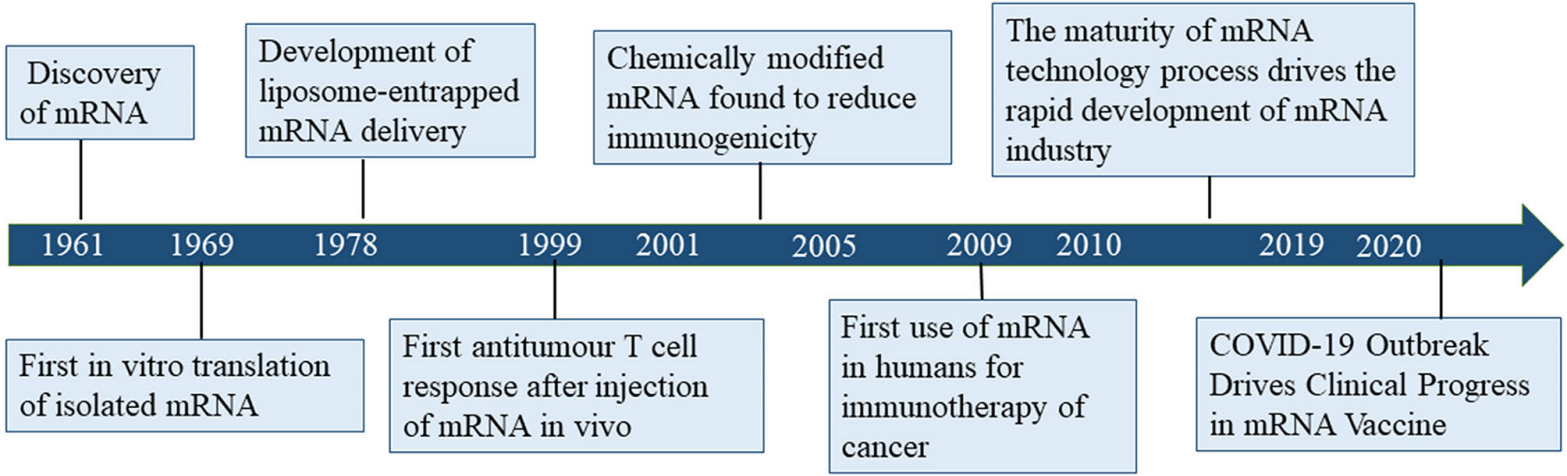
Timeline of mRNA vaccine development.

Despite considerable research effort regarding the development of cancer vaccines, translating cancer vaccines into effective clinical therapies has remained challenging for several decades due to the diversity of tumor antigens and relatively low immune response (3). Since its discovery in 1961, mRNA gradually became the subject of nucleic acid-encoded drug research (**Figure 1**). The concept of nucleic acid-encoded drugs was introduced more than 20 years ago when Wolff et al. demonstrated that intramuscular injections of mRNA produced by *in vitro* transcription (IVT) could express encoded proteins in the muscle at the site of injection. At that time, mRNA had been less well studied than DNA due to its instability, and research was mainly focused on plasmid DNA and viral DNA. During the first decades after the discovery of mRNA, the focus was on determining its structure, function, and metabolism in eukaryotic cells. In the 1990s, IVT mRNA was applied to preclinical exploration as the main component of vaccines for cancer and infectious diseases (4–10).

mRNA vaccines represent an important class of cancer vaccines that are capable of encoding and expressing TAA, TSA, and their associated cytokines. mRNA cancer vaccines can stimulate both humoral and cellular immunity, increasing the adaptability of these vaccines to different diseases and patients. mRNA cancer vaccines have several advantages, including rapid production, flexibility, relatively low cost, and the ability to generate a robust protective immune response. More importantly, from the viewpoint of safety, mRNA does not integrate into the host genome, in contrast to DNA vaccines. Large quantities of accurate and personalized mRNA cancer vaccines can be produced in a short period, making them a promising therapeutic modality. This paper is focused on manufacturing techniques, application, and immunization strategies for mRNA cancer vaccines, and will help us to understand more fully the progress and superiority of these new therapeutic options.

## 2 Strategy for mRNA Vaccine Preparation

**Figure 2:**
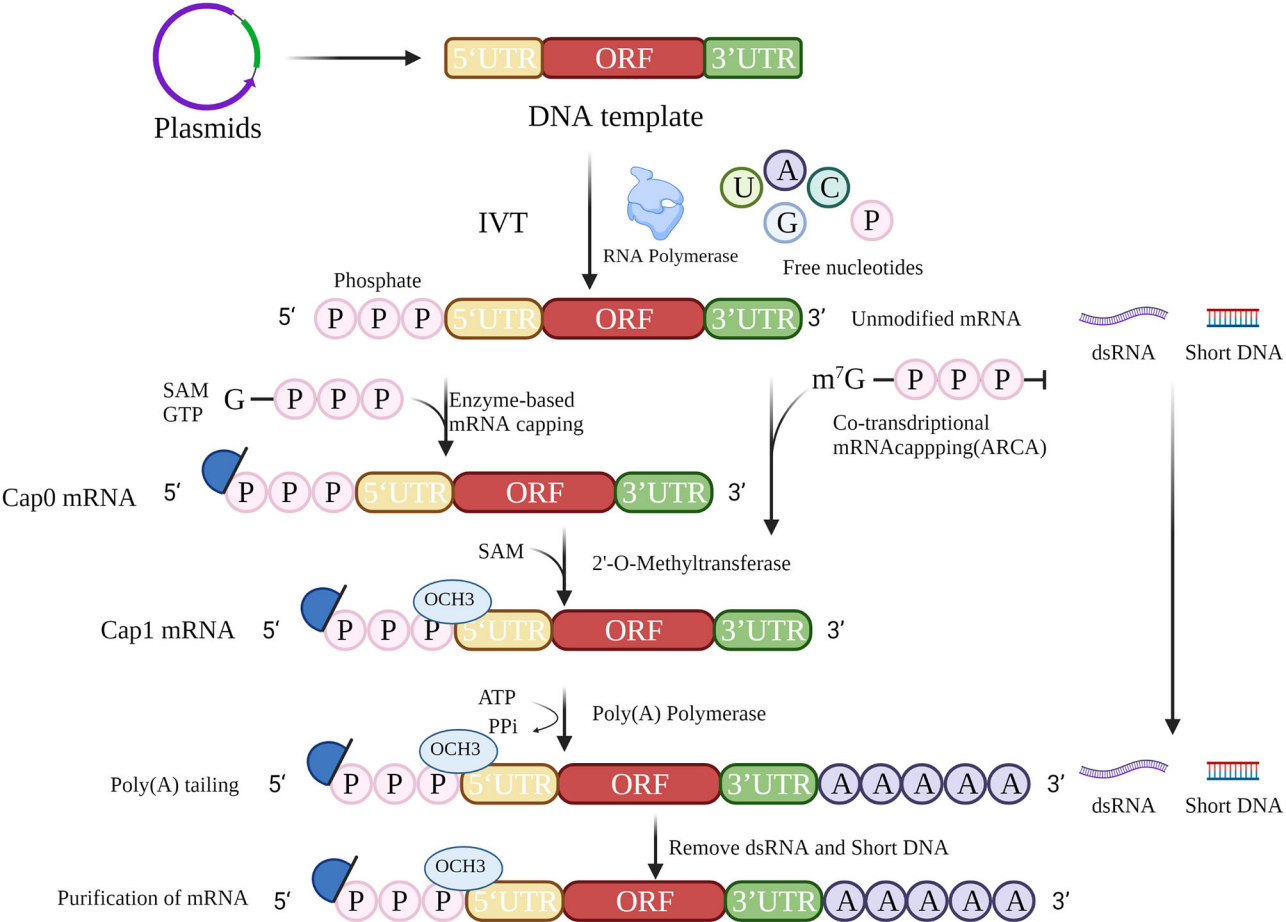
mRNA *in vitro* transcription strategy. The strategy for mRNA preparation consists of template preparation, *in vitro* transcription, 5′ cap addition, 3′ poly(A) tailing, and purification.

The accepted method of mRNA cancer vaccine production involves IVT followed by 5′ capping and polyadenylation at the 3′ end, which resembles the natural process of mRNA maturation in the cytoplasm of eukaryotic cells (**Figure 2**). IVT is a relatively simple process, but the production of high-quality therapeutic mRNAs that do not cause severe inflammation has been a major challenge. Recently, the problems of inflammation and innate immunity have been largely addressed by improvements in capping and tailing techniques, incorporation of modified nucleosides (10) (especially modified uridine), optimization of coding sequences (11), and rigorous purification of IVT mRNA. These techniques will help to reduce the signal of exogenous mRNAs, thus reducing the inflammatory response and improving the translation of mRNAs.

### 2.1 Sequence Construction of mRNA Cancer Vaccines

The mRNA cancer vaccine production process begins with the design of a DNA template for IVT. The template must contain at least the open reading frame (ORF), flanking 5′-untranslated region (5′-UTR), and 3′-UTR. A primer binding site containing an available RNA polymerase recognition site(s) (e.g., T7, T3, or SP6 phage RNA polymerase) (12) is required to initiate IVT.

The target protein encoded by the ORF itself can affect the translation efficiency, and some rare codons can reduce the efficiency. In addition, codon concurrency can be used to optimize the codons corresponding to amino acids and thus improve the efficiency of translation (13). Manipulation of the original sequence, however, may have unfavorable results. In addition, it has been shown that synonymous mutations can be responsible for the occurrence of disease (14).

UTRs have important cellular functions in the regulation of protein expression as well as in influencing the rates of degradation and translation of mRNA. In addition, these functions can be achieved through interactions with different RNA-binding proteins (15). The RNA polymerase binding sites in 5′-UTRs play a vital role in the initiation of translation and formation of preinitiation complexes. In addition, 5′-UTRs facilitate stabilization of mRNA. Shortening the length of the 3′-UTR can improve efficiency of translation, e.g., the 3′-UTRs from α- and β-globins can be used (16). miRNA binding sites can also be included to regulate the expression of target proteins in different tissues or organs, e.g., miRNA-122 binding sites can reduce hepatotoxicity by decreasing expression in normal liver tissues (17). In addition, higher GC content and lower U content not only help to optimize the stability but also reduce the immunogenicity of RNA (18). In conclusion, it is necessary to design an mRNA template sequence with high stability and that can facilitate efficient translation.

### 2.2 *In Vitro* Transcription

IVT is the process of transcribing a designed template DNA strand to an RNA strand according to the complementarity base pairing rule or Chargaff’s rule. Transcription starts after recognition of the promoter by RNA polymerase. During IVT, modified nucleotides are normally applied to reduce immunogenicity (19), including use of pseudouridine (ψ), N6-methyladenine (m6A), 5-methylcytosine (m5C), 2-thiouracil (s2U), and 5-methyluracil (m5U). In particular, m5C and ψ have been reported not only to reduce the immunogenicity of RNA after transcription *in vitro* but also to improve the efficiency of translation.

Synthetic single-stranded RNA contains a 5′ end, a coding region, and a 3′ end. It is then capped and tailed to a mature mRNA molecule, simulating the natural formation process in cells.

### 2.3 5’ Cap Addition

*In vitro* posttranscriptional RNA has a highly immunogenic 5′ triphosphate fraction, which is recognized in the cytoplasm by the pattern recognition receptor (PRR) and then elicits a type I interferon (IFN I) response (20). To prevent the RNA from being identified as exogenous nucleotides, the triphosphate should be removed and a 5′ cap added.

In eukaryotic cells, the typical 5′ cap structure is an inverted 7-methylguanosine (m7G), which is usually co-transcribed with the first nucleotide of RNA *via* a 5′–5′ triphosphate bridge. This 5′ m7G cap or m7Gppp is commonly referred to as “Cap 0”. The 5′ cap structure is essential for initiation of translation, and forms the preinitiation complex by recruiting eukaryotic translation initiation factor 4E (EIF4E). Ribosomes initiate transcription by identification of preinitiation complexes. In addition, the 5′ cap structures increase the stability of RNA and eliminate its immunogenicity.

A 5′ cap can be added in several ways (21). For example, a cap can be achieved by adding a cap analog to the reaction for co-transcription. However, the addition is likely to be incorrectly bound, causing the mRNA to be untranslatable, while anti-reverse cap analog (ARCA) allows the polymerase to add to the nucleotide strand in the correct orientation (22). Posttranscriptional capping, such as Cap 0, is accomplished by removing adenosine triphosphate with phosphatase and adding an m7G cap with 2′-O-methyltransferase. Cap 0 is methylated to Cap 1 *via* methyltransferases to reduce immunogenicity. Neither transcriptional nor posttranscriptional capping guarantees that all RNA molecules will be capped. Incorrect capping activates the PRR leading to increased immunogenicity of exogenous mRNAs (23). Therefore, the success of capping is related to the stability of translation and the immunogenicity of the exogenous mRNA. Further investigations are needed to determine ways to detect the capping success rate.

### 2.4 Poly(A) Tails

The poly(A) tail influences the efficiency and stability of mRNA translation. Poly(A) tails are usually added after transcription with poly(A) polymerase (24) or can be obtained from direct transcription. The poly(A) tail slows down the degradation of RNA by RNA exonucleases, which in turn improves the stability of mRNA. Removal of the poly(A) tail is the first and rate-limiting step in the degradation of most eukaryotic mRNAs. The poly(A) tail is generally 100–250 nucleotides in length, but the optimal length depends on the target cell type. The influenza vaccine studied by Pardi et al. (25) has a poly(A) tail of 101 nt, while the patent disclosed by BioNTech indicates that a poly(A) tail of 120 nt has the highest stability and translation efficiency. Modified adenosine could further protect the poly(A) tail from degradation by ribonucleases (26). The enzymatic reaction conditions, such as temperature and enzyme quality, can influence the length of the poly(A) tail. Consequently, in most clinical trials, the tails in mRNA are generally taken to be of minimum length. Adding oligo(dT) in the DNA template allows better manipulation of the precise length of the tail while, as a part of the template, its length is often limited.

### 2.5 Purification

To ensure the translation of mRNA and the successful expression of the encoded protein, mRNA must be purified to exclude abnormal, truncated, and degraded products. Clinical purification of mRNA by chromatographic techniques removes shorter template fragments produced due to transcription failure and double-strand RNA (dsRNA) generated by self-complementary extension, both of which are common causes of impurities. An alternative method for removing dsRNA from IVT mRNA has been proposed based on adsorption to cellulose (27). During IVT, the amount of dsRNA can be minimized by decreasing the Mg2+ concentration or performing the process at high temperatures. High performance liquid chromatography (HPLC) can be used for relatively comprehensive removal of dsRNA (28), but is costly and has low yield (< 50%). Baiersdorfer et al. recently reported a rapid and inexpensive purification method using selective binding of dsRNA to cellulose in a buffer containing ethanol to remove at least 90% of dsRNA contaminants (27). It has been shown that, using standard techniques, completing mRNA translation and protein expression does not require modified nucleotides and is not dependent on the length of the mRNA, but depends more on the purity and sequence composition (29). Therefore, purification is important for the efficacy of mRNA vaccines. Technology for preparing mRNA is relatively mature, and it will be possible to develop mRNA vaccines rapidly in large quantities at low cost.

## 3 mRNA Delivery Systems

A major challenge in mRNA therapy is the need for effective delivery systems. It is difficult for naked mRNA to transit across the cell membrane because of its dense negative charge and large size. In addition, mRNA itself is an unstable molecule that can be easily degraded (30). Therefore, it is necessary to develop appropriate delivery systems. Traditional methods, such as *in vitro* loaded dendritic cells (DCs), conjugated polymer delivery, and mechanical methods (gene gun, electroporation) have been used to deliver naked mRNA for vaccination (12). However, these methods are complex, expensive (*in vitro* loaded DCs), or not applicable to humans (conjugated polymer delivery, electroporation). The ideal delivery system would protect the mRNA from degradation and enhance efficient cellular uptake. Below, we list several novel delivery methods, including lipid nanoparticles (LNPs), as well as adverse events associated with delivery materials.

### 3.1 Lipid Nanoparticles

LNPs are delivery platforms mainly based on cholesterol, ionizable lipids, and polyethylene glycol (PEG) derivatives (31). Ionizable lipids are amphiphilic structures with hydrophilic head groups, including ionizable amines, hydrocarbon chains that enhance self-assembly, and linkers connecting the head groups to the hydrocarbon chains. In the acidic endosome microenvironment where LNPs are delivered intracellularly, the positive charged lipid interacts with the endosomes to promote membrane fusion and destabilization. This process drives the release of mRNA with LNPs from the liposome. In addition to ionizable lipids, phospholipids and cholesterol are incorporated to enhance the stability of the lipid bilayer and to assist membrane fusion and endosome segregation. Incorporation of lipid-anchored PEG can reduce macrophage-mediated clearance. More importantly, lipid-anchored PEG helps to prevent particle aggregation and improves stability in storage (32).

The current mRNA vaccines approved by the U.S. Food and Drug Administration (FDA) are based on LNP delivery platforms, which match or exceed the effectiveness of other vaccines in both cell-mediated and humoral immunity (33, 34). The first two approved mRNA-based SARS-CoV-2 vaccines demonstrated convincing efficacy in protecting against COVID-19 (35–39). However, there are increasing reports of LNP-induced side effects, such as pain, redness and fever (40). Recent preclinical studies on LNP inflammation indicated that LNPs are inflammatory in some animal models. Intradermal injection results in a rapid and intense immune response characterized by large amounts of neutrophil infiltration and high levels of cytokines. Intranasal administration of LNPs at the same doses resulted in a similar inflammatory response in the lungs as well as high mortality (41). Therefore, it is crucial to develop more effective and safer delivery systems.

### 3.2 Self-Assembled Polymeric Micelles

The self-assembled polymeric micelle delivery system involves a polyethyleneimine copolymer (PVES) modified with vitamin E (VE, α-tocopherol) succinate. VE is on the FDA list of inactive substances for intravenous, oral, and topical usage, and has been utilized as a human immune supplement, as an adjuvant component of emulsions in a variety of veterinary vaccines, and as an adjuvant used in the H1N1 pandemic influenza vaccine (42, 43). VE is also used as a drug delivery vehicle in tocopherol polyethylene glycol succinate (TPGS) micelles (44, 45). In 2016, Liu et al. developed vitamin E-labeled polyethyleneimine (PEI) for gene delivery. Their research showed that VE-labeled PEI markedly enhanced the cellular uptake of plasmid DNA with low toxicity (46). PEI is a water-soluble cationic polymer and VE is a hydrophobic molecule. The binding of VE to PEI results in the formation of a conjugated polymer that can self-assemble into stable micelles (47, 48). PVES micelles showed high transfection efficiency in four cell lines, i.e., HEK-293T, HeLa, Vero, and DC2.4 cells, without significant cytotoxicity. The PVES/mRNA vaccine was shown to trigger antigen-specific CD8+ T cells (Th1 cells) expressing the type 1 immune response cytokine, IFN-γ, and CD4+ T cells expressing the type II cytokine, IL-4 (49). They also assessed the safety of the mRNA vaccine with PVES as a vector. No local inflammatory reactions or other adverse effects at the injection site were observed during the observation period after immunization (49).

### 3.3 Nano-hydrogel

An intelligently responsive nano-DNA hydrogel (nano-hydrogel) was developed as a vehicle to deliver mRNA into cells and stably express proteins. A pH-responsive i-motif cross-linked mRNA scaffold in the shape of an “X” was generated using the well-designed DNA scaffold. The i-motif is a unique DNA quadruplex structure formed by inserting two cytosine-rich duplexes into each other in an antiparallel manner only in an acidic environment. It has been reported that in an acidic microenvironment (50, 51), nano-hydrogels can be internalized by cells to form an i-motif, which is decomposed in lysosomes (52, 53), and the mRNA is then released into the cytoplasm to express the encoded protein. These systems include no chemical agents, so the structure is biocompatible. It is stable because it remains intact outside the cell and only breaks down at low intracellular pH. The results showed that nano-hydrogels have better biocompatibility and higher mRNA expression efficiency than commercial liposomes. Nano-hydrogels represent a promising viable alternative for delivering functional mRNAs *in vivo* because of their good biocompatibility and stability (54). From a safety perspective, the cytotoxicity of liposomes was significantly higher than that of nano-hydrogels with drug loading of Gluc mRNA > 3.18 µg. These results demonstrate the safety of nano-hydrogels for mRNA delivery and suggest that they have promise for applications where it is necessary to deliver large amounts of mRNA (54).

### 3.4 Metal Nanoparticles

Metal nanoparticles (MNPs) are representative of inorganic NPs. In several studies, dendrimers have been used to stabilize MNPs (55–58). Safe and efficient non-viral gene delivery systems can be produced by the combination of MNPs and cationic dendrimers. MNPs, such as gold, are commonly used due to their simplicity, biocompatibility, favorable surface/volume ratio, modifying capability, and low cytotoxicity (59). Mbatha et al. applied folic acid (FA)-modified, poly-amidoamine-generation-5 (PAMAM G5D)-grafted gold NPs (AuNPs) for mRNA delivery (60), and reported the formation of nanocomplexes that provided excellent mRNA protection against RNases. A highly organized structure was formed due to electrostatic interactions between negatively charged mRNA and highly cationic PAMAM G5D-containing NPs (61). We also noted that over 80% of the cells were capable of tolerating these nanocomplexes. These AuNPs showed excellent transfection efficiency, suggesting that the dendrimers and AuNPs played significant synergistic roles in the process. This study further confirmed that the main pathway into receptor-positive cells was mediated by the folate receptor, and that the transfection level of FA receptor-positive cell lines was significantly higher than FA receptor-negative cell lines.

### 3.5 Adverse Events Related to mRNA Delivery Materials

Adverse allergic reactions to mRNA vaccines are rare, but a few still produce severe reactions. All mRNA delivery systems subjected to clinical trials to date are based on LNPs, and the exact compositions of delivery systems for two SARS-CoV-2 mRNA vaccines (mRNA-1273 and BNT162b2) authorized for emergency use in the COVID-19 pandemic have been publicly disclosed. The LNPs encapsulating the mRNA vaccine contain PEG2000, which is the main cause of allergic reactions associated with mRNA vaccines. As excipients of drugs, PEG components are thought to be a risk factor for IgE-mediated responses and recurrent severe allergic reactions. The risk of sensitization to drugs containing high molecular weight PEG appears to be high, and clinical contrast agents have been reported to induce severe allergic reactions after bowel preparation with drugs containing PEG3350 and higher molecular weight PEGs. In addition, doxorubicin liposomes containing PEG were also reported to produce allergic reactions.

Treatment of tumors with mRNA vaccines often requires repeated administration, so there is a concern that slow degradability of delivery materials may accumulate and have the potential to cause toxicity in the liver. For example, MC3 with a dilinoleic alkyl tail in LNPs is such a material. In a study by Moderna, lipid H or SM-102 was found to be the best intramuscular substitute for MC3.

The role of mRNA delivery materials involves safely and effectively delivering mRNA into cells as well as reducing associated adverse events, such as immune rejection and liver toxicity. With the identification of several adverse effects, the development of safe delivery systems and simulation of biological natural delivery methods have become areas of active research, and include the use of MNPs, nano-hydrogels, self-assembled polymeric micelles, bio-inspired nanovehicles, etc.

## 4 How mRNA Works on the Immune System

After injection of the mRNA cancer vaccine, the mRNA-encoded protein is synthesized by the ribosome and then posttranslationally modified to produce a correctly folded functional protein, which is presented to the immune system. The process is similar to the natural process of RNA virus infection and successive induction of a protective immune response. The entry of exogenous mRNA into the cytoplasm results in a similar reaction to that of endogenous mRNA. After mRNA is translated into proteins in the cytoplasm, the proteins are modified and enter subcellular compartments, such as the secretory pathway, cell membrane, nucleus, mitochondria, or peroxisomes, through targeting sequences or transmembrane structural domains (62). Therefore, delivery of exogenous mRNA into the cytoplasm is essential for antigen expression.

### 4.1 mRNA Cancer Vaccine Induces an Innate Immune Response

#### 4.1.1 Immune Cell Recognition

**Figure 3:**
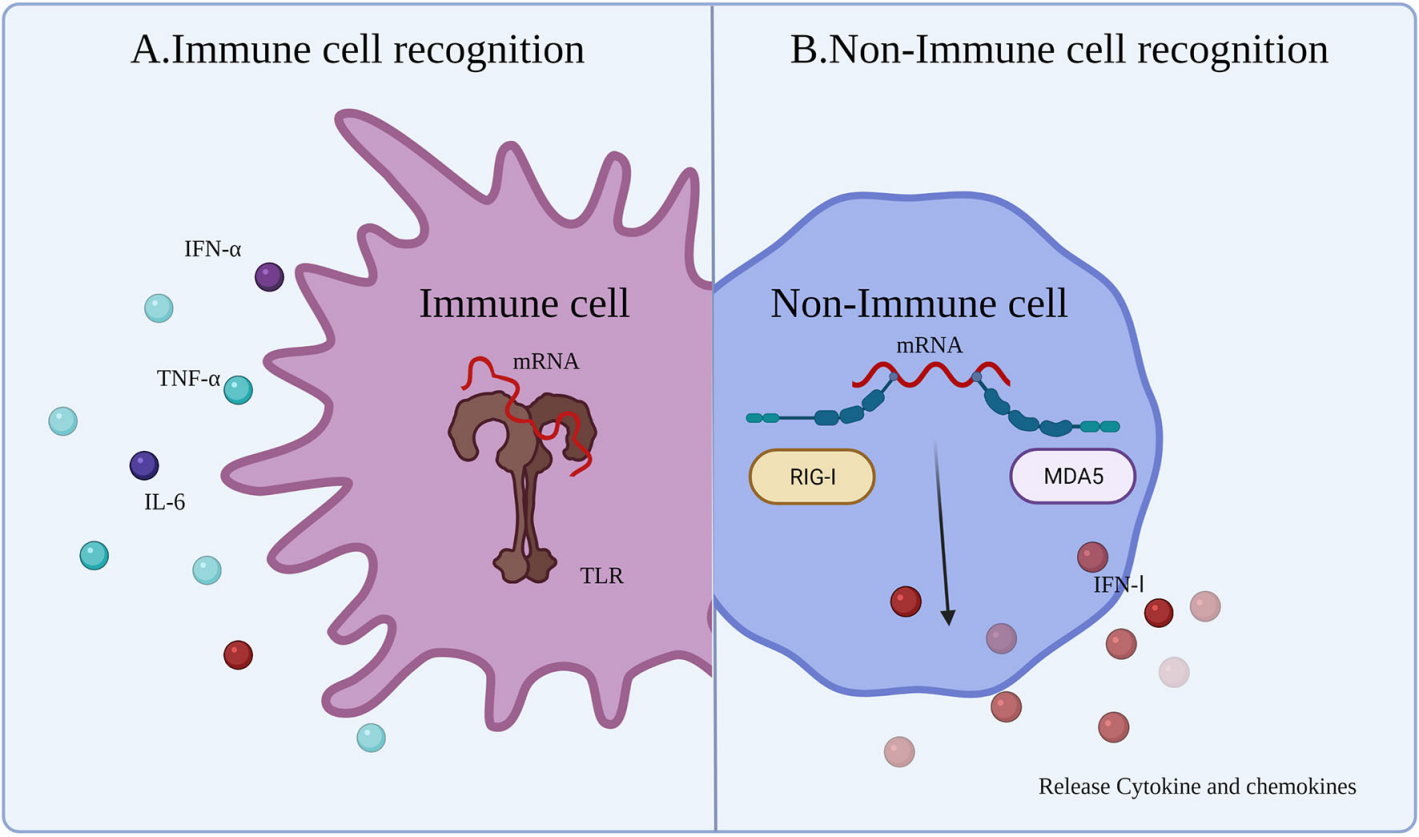
Innate immune response to mRNA vaccine. After the exogenous mRNA enters the human body, it produces an innate immune response. **(A)** The response occurs mainly in the immune cells. Immune responses are activated though TLRs to detect PAMPs (exogenous mRNA). **(B)** The response occurs mainly in non-immune cells. RIG-1 and MDA5 sense the exogenous mRNA and then induce an IFN I response.

Innate immune responses are activated by the host immune system through PRRs detecting pathogen-associated molecular patterns (PAMPs) (**Figure 3A**) (63). After injection of the vaccine, the mRNA and delivery system components will be identified as exogenous substances by a series of PRRs leading to activation of Toll-like receptors (TLRs), such as TLR3, TLR7, and TLR8, mainly expressed on antigen-presenting cells (APCs). Exogenous IVT mRNA can be recognized by various PRRs on the cell membrane, endosomes, as well as in the cytoplasm, and has a role in stimulating the intrinsic immune response (64). TLRs, which are recognized as PRRs, play a role in detecting PAMPs. APCs recognize mRNA and activate TLRs, which sense PAMPs and initiate an innate immune response (65), producing proinflammatory cytokines and co-stimulatory molecules on APCs (e.g., DCs) (66). This ultimately aids in the production of adaptive B cell and T cell responses (65).

The immunogenicity of IVT mRNA is mainly mediated by TLR7 and TLR8. TLR7 is expressed by B cells (62), macrophages, and DCs (67), and mediates the process of detecting single-stranded RNA (ssRNA). Activation of B cells is mediated by the myeloid differentiation marker 88 (MYD88)/TLR7-dependent signaling pathway, providing stimulation to upregulate the mRNA vaccine-induced adaptive immune response. In addition, TLR7 signaling also increases the generation of proinflammatory cytokines and antigen presentation, as well as the improvement of memory B cell survival (68). Furthermore, the MYD88 pathway can upregulate the IFN I response and induce a proinflammatory state by secreting cytokines (63, 69). In the cytoplasm, some other PRRs can sense different types of RNA, such as dsRNA and ssRNA (63). dsRNA can be generated during the process of IVT. IVT mRNA doped with dsRNA can upregulate and activate protein kinase R (PKR) and oligoadenylate synthetases (OAS). Subsequently, mRNA degradation occurs by the IFN I-mediated immune response. Activation of multiple PRRs and production of IFN I may be beneficial or detrimental for anticancer immunotherapy. The beneficial effects are because activation of immune factors and maturation of immune cells contribute to the function of subsequent mRNA-expressed protein. The detrimental effects are that mRNA would be degraded or translation blocked by innate immunity.

#### 4.1.2 Non-immune Cell Recognition

In non-immune cells, cytoplasmic retinoic acid-inducible gene I-like receptor (RLR) and melanoma differentiation-associated gene 5 (MDA5) sense exogenous mRNA and regulate the generation of cytokines and chemokines (24), resulting in innate immune cell recruitment to the mRNA injection site (70). Although early induction of strong cytokine production is advantageous for improving vaccine efficacy, cytokines can lead to severe side effects, including autoimmunity, or weaken the immune response to the mRNA vaccine making the antitumor immune effects of the cancer vaccine incomplete. Therefore, different approaches have been sought in mRNA vaccine technology to minimize the induction of cytokines, such as IFN I. Miao et al. (20) reported that mRNA incorporating unsaturated lipid tails, dihydroimidazole junctions, and cyclic amine head motifs could activate APCs through the intracellular interferon gene (71) pathway rather than the TLR pathway. The effects of APC activation by the STING pathway can reduce the expression of cytokines, reducing the side effects of the cytokine-induced autoimmune response and improving the antitumor effect (**Figure 3B**).

In summary, innate sensing of exogenous mRNA may lead to mRNA translational arrest, mRNA degradation, and sequential secondary antigen-specific immune responses (72), suggesting a close link between innate and acquired immunity after mRNA inoculation.

### 4.2 Induction of Acquired Immunity From mRNA Cancer Vaccine

#### 4.2.1 Antigen Presentation

**Figure 4:**
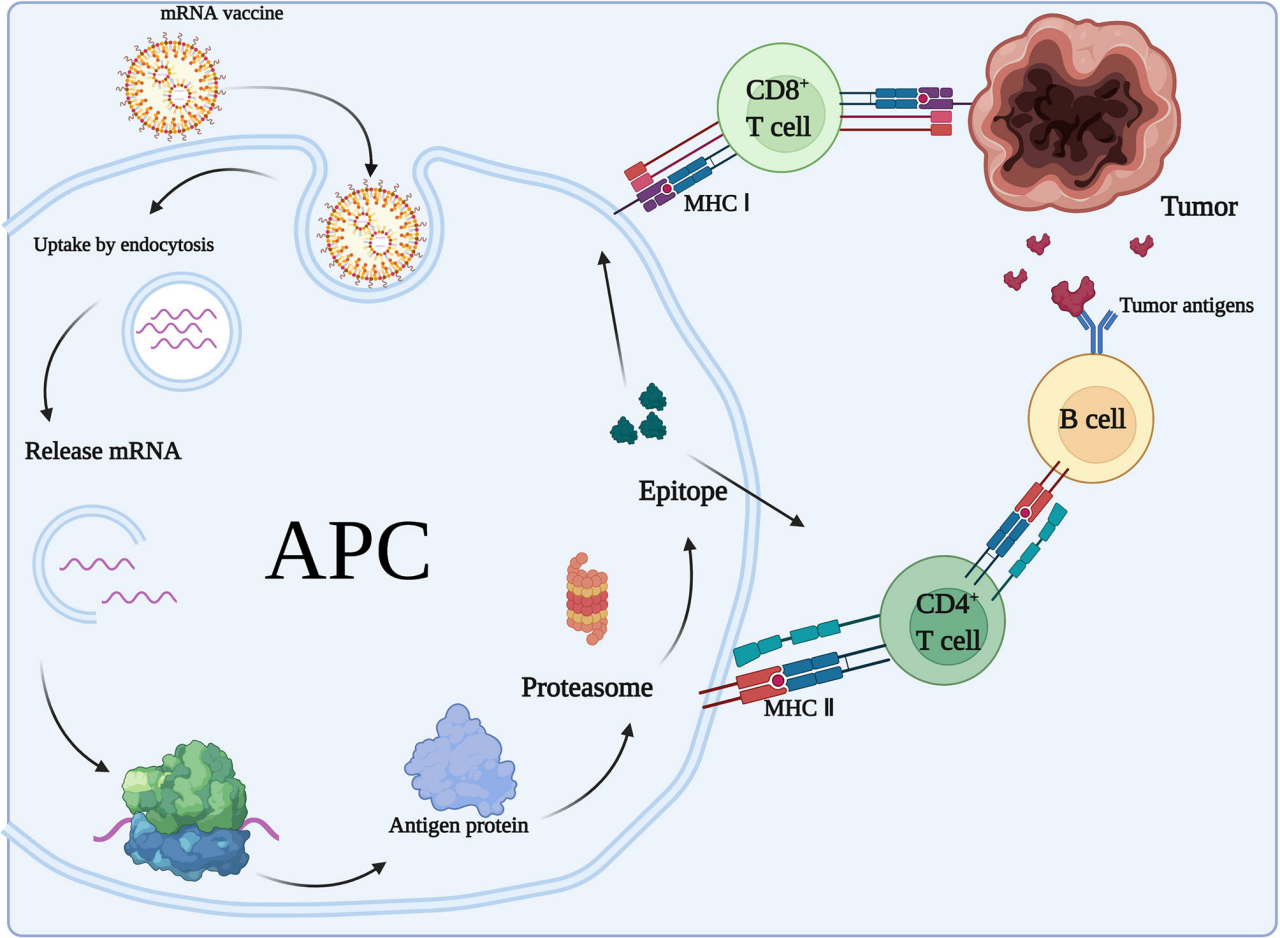
Adaptive immune response to mRNA vaccines. In the case of mRNAs encoding antigens, mRNA vaccines exert immunological effects mainly through adaptive immune responses. After mRNA vaccination, the encoded proteins will be translated and taken up by APCs, which present the antigens to CD4+ T cells *via* MHC II and cross-present them to MHC I on CD8+ T cells. CD4+ T cells can enhance the antitumor effects of B cells.

After mRNA vaccination, the encoded proteins will be translated and presented to the immune system and stimulate acquired immunity (**Figure 4**). The mRNA-encoded proteins are translated and taken up by APCs (e.g., DCs) through microphagocytosis, endocytosis, or phagocytosis (73). They may form phagocytic vesicles or endosomes containing antigenic proteins (74) that are presented through major histocompatibility complex I and II (MHC-I and MHC-II, respectively) on DCs. APCs can present exogenous antigens to CD4+ T cells *via* MHC-II and cross-present them to MHC-I on CD8+ T cells. The resulting induction of cytotoxic T lymphocytes is called cross-excitation. CD4+ T cells can enhance the antitumor effects of B cells and CD8+ T cells by secreted cytokines (75). Finally, target cell clearance mediated by antigen-specific B cells and T cells occurs due to clonal expansion. In addition, all nucleated cells have the ability to process mRNA and present translated proteins or peptides on MHC-I. However, only APCs present antigens on MHC-I and MHC-II, thereby inducing an immune response from CD4+ T cells and B cells. In addition, DCs can internalize the cytoplasmic and cell membrane material of living cells to initiate T cell responses (76).

#### 4.2.2 Antigen Classification

Specific antigen presentation is needed to induce acquired immunity. mRNA cancer vaccines usually encode TAAs that are expressed on cancer cells. These TAAs can be further divided into tissue differentiation antigens, such as human carcinoembryonic antigen (77) or MART-1, which are also expressed in healthy tissues; tumor germline (testicular cancer) antigens (e.g., NY-ESO-1 or MAGE-3); tumor cell-overexpressed normal proteins (e.g., EGFR, MUC1, Her2/neu); viral proteins (e.g., EBV, HPV); and tumor-specific mutational antigens (e.g., MUM-1, β-catenin or CDK4) (78, 79). Genetic abnormalities drive tumor development (80). Somatic mutations may generate neoantigen epitopes, tumor-derived peptides that can bind to MHC (81) and be recognized by T cells. Therefore, mRNA vaccines encoding neoantigens are considered to be the best cancer vaccine candidates (80). Similarly, T cells that recognize these antigens mediate clinical responses after metastasis or immune checkpoint suppression (82). Moreover, it has been shown that tumors containing higher epitope loads of neoantigens, such as melanoma, non-small cell lung cancer (NSCLC), and mismatch repair-deficient colorectal cancer (CRC), respond better to immune CPIs (83).

#### 4.2.3 Immunological Effects

Peptide-based vaccines are MHC-restricted, whereas mRNA vaccines allow the combination of mRNAs encoding different antigens. mRNA-electroporated DCs have multiple MHC-I and II-restricted peptides and induce polyclonal CD4+ and CD8+ T cell responses. CD4+ helper T (Th) cells are essential for the effective induction of B cell and cytotoxic T lymphocyte (CTL) responses (83). mRNA vaccines further reinforce the immune response in the presence of helper epitopes. CD4+ T cell responses after the introduction of mRNA into DCs are mediated by autophagy (84). Finally, vaccine compositions can include mRNAs encoding immunomodulatory proteins, which can further enhance their efficacy. In summary, mRNA vaccines encoding two or more proteins or long peptides can achieve an extensive polyclonal immune response, avoiding restriction to MHC molecules and immune escape caused by antigen loss.

Application of mRNAs encoding mutant antigenic epitopes produces intensive antigen-specific CD8+ T cell responses and effective and durable CD4+ T cell-mediated tumor regression. Kreiter et al. demonstrated that most tumor-specific mutations are recognized by CD4+ T cells, which have intensive antitumor activity. The CD4+ T cell response is dominated by the Th1-based immune response as well as interferon-γ produced by CD4+ and CD8+ T cells (85, 86). For this strong Th1 response, different studies attempted to regulate Th differentiation using mRNA vaccines.

In addition to inducing T cell immunity, mRNA vaccines also induce neutralizing antibodies. T follicular helper (Tfh) cells are not only essential for germinal center development, but also drive immunoglobulin-like conversion, affinity maturation, and durable B cell memory responses. Although the precise mechanism of action of Tfh is not yet known, such cells are activated by mRNA vaccines, which produce sufficient numbers of potent and durable neutralizing antibodies (87). Pardi et al. (77) used LNP-encapsulated mRNA vaccines for subcutaneous administration and found them to induce efficient neutralizing antibodies. Sustained antigen expression resulted in high antibody titers as well as germinal center B cell and Tfh responses (88).

## 5 Clinical Development of mRNA Cancer Vaccine

IVT mRNA-based vaccines are gradually being developed for a variety of tumor treatments. Currently, mRNA cancer vaccines are classified as encoding TAAs, TSAs, cytokines, and antibodies based on the final product types. In most clinical trials, mRNA cancer vaccines have been applied to treat aggressive, refractory, and metastatic tumors. Here, we summarize the clinical trials in different cancer treatments based on product types (**Tables 1**–**3**). Moreover, we discuss therapeutic regimens to explore the possibility of application of mRNA vaccines for various cancers.

**Table 1:** Clinical trials of mRNA encoding TAAs.

Antigen	Brand		Title	Conditions	NCT Number	Phase	Study Start	Status
**NY-ESO-1, MAGE-C3, tyrosinase, TPTE**	BNT111		Trial With BNT111 and Cemiplimab in Combination or as Single Agents in Patients With Anti-PD-1-refractory/Relapsed, Unresectable Stage III or IV Melanoma	Melanoma Stage III/IV	NCT04526899	Phase 2	May 19, 2021	Recruiting
**RBL038, RBL039, RBL-040, RBL-041, RBL-045**	BNT112		PRO-MERIT (Prostate Cancer Messenger RNA Immunotherapy)	Prostate Cancer	NCT04382898	Phase 1/2	December 19, 2019	Recruiting
**HPV16(+) E6、E7**	BNT113		A Clinical Trial Investigating the Safety, Tolerability, and Therapeutic Effects of BNT113 in Combination With Pembrolizumab Versus Pembrolizumab Alone for Patients With a Form of Head and Neck Cancer Positive for Human Papilloma Virus 16 and Expressing the Protein PD-L1	Head and Neck Cancer Recurrent Head and Neck Cancer	NCT04534205	Phase 2	January 7, 2021	Recruiting
**antigen commonly expressed by NSCLC cells**	BNT116		Clinical Trial Evaluating the Safety, Tolerability and Preliminary Efficacy of BNT116 Alone and in Combinations in Patients With Advanced Non-small Cell Lung Cancer	NSCLC	NCT05142189	Phase 1	April 2022	Not yet recruiting
**3 OC TAA**	W_ova1		Ovarian Cancer Treatment With a Liposome Formulated mRNA Vaccine in Combination With (Neo-)Adjuvant Chemotherapy	Ovarian Cancer	NCT04163094	Phase 1	November 25, 2019	Recruiting	
**PSA, PSCA, PSMA, STEAP1**	CV9103		Safety and Efficacy Trial of a RNActive^®^-Derived Prostate Cancer Vaccine in Hormone Refractory Disease	Prostate cancer	NCT00831467	Phase 1/2	January 2009	Completed	
**PSA, PSCA, PSMA, STEAP1, PAP, MUC1**		CV9104	Trial of RNActive^®^-Derived Prostate Cancer Vaccine in Metastatic Castrate-refractory Prostate Cancer	Prostate cancer	NCT01817738	Phase 1/2	August 2012	Terminated
**MAGE-C1, MAGEC2, NY-SEO-1, survivin, 5 T4**		CV9201	Trial of an RNActive^®^-Derived Cancer Vaccine in Stage IIIB/IV Non-Small Cell Lung Cancer(NSCLC)	Stage IIIB/IV NSCLC	NCT00923312	Phase 1/2	May 2009	Completed
**NY-ESO-1, MAGEC1, MAGE-C2, 5 T4, survivin, MUC1**		CV9202	Phase 1/2 Study of Combination Immunotherapy and mRNA Vaccine in Subjects With NSCLC	NSCLC	NCT03164772	Phase 1/2	December 20, 2017	Completed

**Table 2:** Clinical trials of mRNA encoding TSAs.

Antigen	Brand	Title	Conditions	NCT Number	Phase	Study Start	Status
**Colorectal Cancer TSA**	BNT122	A Phase II Clinical Trial Comparing the Efficacy of RO7198457 Versus Watchful Waiting in Patients With ctDNA-positive, Resected Stage II (High Risk) and Stage III Colorectal Cancer	Colorectal Cancer Stage II/III	NCT04486378	Phase 2	March 8, 2021	Recruiting
**Cancer TSAs**	RO7198457	A Study of Autogene Cevumeran (RO7198457) as a Single Agent and in Combination With Atezolizumab in Participants With Locally Advanced or Metastatic Tumors	Melanoma NSCLC Bladder Cancer	NCT03289962	Phase 1	December 21, 2017	Active, not recruiting	
**Cancer TSAs**	RO7198457	A Study of the Efficacy and Safety of RO7198457 in Combination With Atezolizumab Versus Atezolizumab Alone Following Adjuvant Platinum-Doublet Chemotherapy in Participants Who Are ctDNA Positive After Surgical Resection of Stage II-III Non-Small Cell Lung Cancer	NSCLC	NCT04267237	Phase 2	March 31, 2021	Withdrawn	
**Cancer TSAs**	RO7198457	A Study to Evaluate The Efficacy And Safety Of RO7198457 In Combination With Pembrolizumab Versus Pembrolizumab Alone In Participants With Previously Untreated Advanced Melanoma	Advanced Melanoma	NCT03815058	Phase 2	January 8, 2019	Active, not recruiting	
**Cancer TSAs**	mRNA-4157	Safety, Tolerability, and Immunogenicity of mRNA-4157 Alone in Participants With Resected Solid Tumors and in Combination With Pembrolizumab in Participants With Unresectable Solid Tumors	Solid Tumors	NCT03313778	Phase 1	August 14, 2017	Recruiting	
**Cancer TSAs**	mRNA-4157	An Efficacy Study of Adjuvant Treatment With the Personalized Cancer Vaccine mRNA-4157 and Pembrolizumab in Participants With High-Risk Melanoma (KEYNOTE-942)	Melanoma	NCT03897881	Phase 2	July 18, 2019	Active, not recruiting	
**KRAS mutations: G12D,G12V G13D,G12C**	mRNA-5671/V941	A Study of mRNA-5671/V941 as Monotherapy and in Combination With Pembrolizumab (V941-001)	NSCLC Pancreatic Neoplasms Colorectal Neoplasms	NCT03948763	Phase 1	June 26, 2019	Active, not recruiting	
**Cancer TSAs**	SW1115C3	A Study of Neoantigen mRNA Personalised Cancer in Patients With Advanced Solid Tumors	Solid Tumor	NCT05198752	Phase 1	March 12, 2022	Not yet recruiting	
**Cancer TSAs**	IVAC MUTANOME	IVAC MUTANOME Phase I Clinical Trial	Melanoma	NCT02035956	Phase 1	December 2013	Completed	
**Cancer TSAs**	NA	Clinical Study of Personalized mRNA Vaccine Encoding Neoantigen in Patients With Advanced Digestive System Neoplasms	Esophageal Squamous Carcinoma Gastric/ Pancreatic/ Colorectal Adenocarcinoma	NCT03468244	Not Applicable	May 1, 2018	Unknown	
**Cancer TSAs**	NA	Safety and Efficacy of Personalized Neoantigen Vaccine in Advanced Gastric Cancer, Esophageal Cancer and Liver Cancer	Gastric/ Esophageal/ Liver Cancer	NCT05192460	Not Applicable	February 2022	Recruiting	
**Cancer TSAs**	NA	Safety and Efficacy of Personalized Neoantigen Vaccine in Advanced Gastric Cancer	Gastric Cancer	NCT05227378	Not Applicable	March 2022	Not yet recruiting	
**Cancer TSAs**	NA	Clinical Study of Personalized mRNA Vaccine Encoding Neoantigen in Patients With Advanced Esophageal Cancer and Non-small Cell Lung Cancer	Esophageal Cancer Non-Small Cell Lung Cancer	NCT03908671	Not Applicable	May 2019	Not yet recruiting	

NA, Not Applicable.

**Table 3:** Clinical trials of mRNA encoding an immunostimulant.

Immune-stimulant	Brand	Title	Conditions	NCT Number	Phase	Study Start	Status
**IL-12sc, IL-15 sushi, GM-CSF, IFNα**	SAR441000 (BNT131)	A First-in-Human Dose Escalation and Expansion Study to Evaluate Intratumoral Administration of SAR441000 as Monotherapy and in Combination With Cemiplimab in Patients With Advanced Solid Tumors	Metastatic Neoplasm	NCT03871348	Phase 1	January 3, 2019	Recruiting
**Optimized IL-2**	BNT151	BNT151 as a Monotherapy and in Combination With Other Anti-cancer Agents in Patients With Solid Tumors	Multiple solid tumors	NCT04455620	Phase 1/2	January 26, 2021	Recruiting
**IL-7,IL-2**	BNT152, BNT153	Dose Escalation Trial of BNT152+153 in Patients With Cancer	Solid Tumor	NCT04710043	Phase 1	June 8, 2021	Recruiting
**TLR7/8, RIG-1**	CV8102	Study of Intratumoral CV8102 in cMEL, cSCC, hnSCC, and ACC	Skin cancer	NCT03291002	Phase 1	September 25, 2017	Active, not recruiting
**OX40L**	mRNA-2416	Dose Escalation and Efficacy Study of mRNA-2416 for Intratumoral Injection Alone and in Combination With Durvalumab for Participants With Advanced Malignancies	Relapsed/Refractory Solid Tumor Malignancies or Lymphoma Ovarian Cancer	NCT03323398	Phase 1/2	August 9, 2017	Active, not recruiting
**OX40L, IL-23, IL-36γ**	mRNA-2752	Dose Escalation Study of mRNA-2752 for Intratumoral Injection to Participants in Advanced Malignancies	Relapsed/Refractory Solid Tumor Malignancies or Lymphoma	NCT03739931	Phase 1	November 27, 2018	Recruiting
**IL-12**	MEDI1191	A Study of MEDI1191 in Sequential and Concurrent Combination With Durvalumab in Subjects With Advanced Solid Tumors	Solid Tumors Cancer	NCT03946800	Phase 1	May 8, 2019	Recruiting

### 5.1 Clinical Trials of mRNA Encoding TAAs

Appropriate antigen selection is the basis for the development of cancer vaccines. Non-mutant shared tumor antigens are generally selected as targets for mRNA cancer vaccines. A typical example is the melanoma vaccine encoding selected malignant melanoma-associated antigens. In multiple clinical trials, NY-ESO-1, tyrosinase, MAGE-A3, MAGE-C2, and TPTE have been employed as TAAs for melanoma (**Table 1**). BNT111 is a cancer vaccine, a lead candidate for the BioNTech FixVac platform, which utilizes a fixed combination of TAAs designed to trigger a powerful and precise immune response against cancer. The FDA approved the BNT111 Fast Track Designation, a new cancer immunotherapy for advanced melanoma at the end of 2021, and BNT111 is currently under investigation in two clinical trials. A recent report of an exploratory interim analysis from a phase I trial (NCT02410733) showed that BNT111 is a potent immunotherapy in patients with immune CPI-experienced melanoma (89). A subsequent randomized phase II trial (NCT04526899) is aimed at supporting the initial data from a phase I trial by investigating the safety and antitumor responses of BNT111 alone or in combination with Libtayo (cemiplimab), an anti-PD-1 monoclonal antibody. CV9201 (NCT00923312) and CV9202 (NCT01915524, NCT03164772) are two vaccine target antigens expressed in NSCLC (90–92). The clinical trials showed that these two vaccines are well tolerated and immune responses could be detected after treatment, thus supporting further clinical investigation of mRNA-based immunotherapy in NSCLC, including combinations with CTLA-4.

TAAs are attractive vaccine targets but are more suitable for certain solid tumors, such as melanoma and NSCLC, which have TAAs. Moreover, as TAAs are non-mutated self-antigens, they are also present in normal tissues. Vaccines expressing TAAs may trigger both central and peripheral immune tolerance responses, thereby reducing clinical vaccination efficiency observed in many vaccine trials. Therefore, most vaccines expressing TAAs are still used as adjunctive therapy in combination with immune CPIs (**Table 1**).

### 5.2 Clinical Trials of mRNA Encoding TSAs

mRNA vaccines encoding a variety of mutated antigens are ideal for treating mutation-induced malignancies. This type of cancer vaccine has been examined in the greatest number of clinical trials. With the development of next-generation sequencing (NGS) technology, personalized mRNA cancer vaccines encoding mutated antigens can be produced to stimulate the immune system, to identify and kill cancer cells. There is a great deal of clinical and research interest tin such personalized cancer vaccines. Two personalized mRNA cancer vaccines are currently in phase II clinical trials, Moderna vaccine mRNA-4157 and BioNTech vaccine BNT122 (RO7198457). mRNA-4157 was specifically screened and encoded 20–34 neoantigens on a single mRNA molecule, depending on the patient’s cancer mutations. Interim data from a phase I trial showed that mRNA-4157 monotherapy or in combination with the PD-1 inhibitor Keytruda (NCT03313778) was well tolerated at all doses tested and triggered a neoantigen-specific T cell response. Due to the positive phase I trial results, personalized cancer vaccine mRNA-4157 is now in a phase II clinical trial (NCT03897881) to evaluate whether postoperative adjuvant therapy with mRNA-4157 and pembrolizumab improves recurrence-free survival compared to pembrolizumab alone in patients with complete resection of cutaneous melanoma and a high risk of recurrence. BioNTech explored the efficacy and safety of RO7198457 in combination with PD-L1 antibody to treat various cancers. Phase II clinical trials in melanoma, NSCLC, and CRC were initiated in the first half of 2021.

Personalized mRNA vaccines provide a new direction for precision tumor treatment. With existing clinical data and a large number of ongoing clinical trials, personalized mRNA vaccines have potential as adjuvant therapy for immunotherapy. However, the data demonstrating efficacy were generally in completely individualized immune responses. In addition, these tumor-specific immune responses rarely translate to tumor reduction. Overall, mRNA cancer vaccines encoding TSAs can improve the tumor immunogenicity and increase the sensitivity of tumor cells to immune CPIs, so the clinical response rate is meaningful.

### 5.3 Clinical Trials of mRNA Encoding Immunostimulants

In theory, mRNA cancer vaccines could encode any protein, including immunostimulants, which could reshape the tumor immune microenvironment (TIME) (93), complement immune CPIs, and overcome tumor immune tolerance. This has become an important direction for mRNA cancer vaccine research. Over the last several years, clinical trials of mRNAs encoding cytokines have been conducted. At present, there are only seven product candidates in clinical trials, which belong to Moderna and BioNTech. Moderna developed the first clinical trial expressing mRNA-encoded immunostimulant (mRNA-2416: mRNA-encoded OX40L, NCT03323398) in 2017. This study assessed the safety and tolerability of escalating doses of mRNA-2416 alone and in combination with durvalumab in patients with advanced malignancies. The data presented at the American Association for Cancer Research (AACR) showed that mRNA-2416 monotherapy had been tolerable at all dose levels, with no reported dose-limited toxicity, and the majority of related adverse events were grade 1 or grade 2. More importantly, the observations of broad proinflammatory activity and beneficial changes in the TIME with upregulation of PD-L1 support the evaluation of combination of intratumoral mRNA-2416 with the anti-PD-L1 inhibitor, durvalumab, in solid tumors (94). This intratumoral injection of mRNA-2416 has entered a clinical phase II trial for advanced ovarian carcinoma. A dose escalation study (mRNA-2752: OX40L/IL23/IL36γ, NCT03739931) of intratumoral injection of triplet mRNA vaccine was carried out in patients with advanced malignancies. Early results showed that mRNA-2752, administered in combination with the anti-PD-L1 antibody, durvalumab, was well tolerated at all doses and showed signs of antitumor activity. BioNTech developed BNT131 encoding IL-12sc, IL-15sushi, GM-CSF, and IFNα as monotherapy and in combination with cemiplimab in patients with advanced solid tumors. Sanofi named the candidate SAR441000, and registered a clinical trial (NCT03871348) in 2019. SAR441000 was generally well tolerated both as monotherapy and in combination with cemiplimab. An immunomodulatory effect was mediated by downstream effector cytokines and T cell infiltration (95).

There are still serious clinical limitations for systemic administration of cytokine-based mRNA vaccines. For example, the short half-life requires frequent administration, leading to dose-limiting toxicity. Existing preclinical studies demonstrated that the toxic side effects of systemic administration can be avoided by intratumoral administration (96, 97). In addition, the main active site of immunostimulants is in the TIME, so clinical studies have mainly adopted intratumoral injection. To date, tumor vaccines encoding immunostimulants have been shown to be effective as adjuvants to tumor immunotherapy.

## 6 Safety and Prospects for mRNA Vaccines

Until 2020, no single mRNA vaccine regimen had been approved globally. Due to the COVID-19 pandemic, the FDA approved different mRNA vaccines against SARS-CoV-2, demonstrating the advantages of rapid and effective production of mRNA vaccines against emerging infectious diseases. However, unlike infectious disease vaccines that target well-defined antigens for prophylactic vaccination, most tumor-targeting antigens exhibit a high degree of interindividual heterogeneity and are limited in number and poorly characterized, raising issues about the safety and efficacy of mRNA cancer vaccines. Although anticancer preventive vaccines are still in preclinical studies, their clinical translation is limited by the difficulty of antigen prediction and poor immunogenicity. In addition, most antigens for infectious diseases (bacterial or viral) are exogenous motifs presented by MHC II molecules, and vaccines against these exogenous antigens induce neutralizing antibody-mediated humoral responses. In some cases, CD4+ T cell-mediated immune responses are partially involved and required, while CD8+ cytotoxic T cells play a key role in the clearance of malignant cells bearing somatic mutations. Therefore, therapeutic anticancer vaccines need to enhance not only the humoral CD4+ T cell response but also activation of the MHC I-mediated CD8+ T cell response, which further increases the difficulty of effectively achieving strong antitumor immunity (98). Another major obstacle in the development of a highly effective anticancer vaccine is the identification and efficient delivery of highly immunogenic TSAs. Tumors vary across different individuals and some are less immunogenic and can evade recognition by the host immune system. Even if the antigen is immunogenic, the suppressive TIME can prevent effective T cell infiltration, leading to T cell depletion (98). Therapeutic cancer vaccines would require higher and multiple repeated doses, so higher safety standards are necessary for mRNA production (62).

The safety of mRNA vaccines is reflected by the ability of mRNA cancer vaccines to encode multiple antigens simultaneously as well as being non-integrating, highly degradable, and have no insertional mutagenic potential. The mRNA produced by IVT is free of cellular and pathogenic viral components and has no potential for infection; most mRNA vaccines tested in ongoing clinical trials are usually well tolerated with few injection site-specific immune reactions (99). Systemic inflammation can also limit the innate immune response to the local injection site by removing contaminants, such as dsRNA, or by changing the route of administration. As activation of the IFN I response may be related to autoimmunity, the risk of increased autoimmune response in patients must be assessed before mRNA vaccination (12). Rapid production is another advantage of mRNA cancer vaccines, and the maturity of mRNA manufacturing techniques allows the production of novel vaccines in a short time. The recent discovery and identification of new antigens have facilitated the development of personalized vaccine therapies. Several clinical studies performed by BioNTech and Moderna have demonstrated potent antitumor immunity using personalized vaccines in some clinical trials for treatment of multiple solid tumors, initiating a new era of therapeutic oncology vaccines. To improve the anticancer efficacy of mRNA vaccines further, specific adjuvants, immune CPIs, T cell-activated monoclonal antibodies, modulation of the TME, or combination with radiation therapy or chemotherapy should be used to avoid immune escape and thus improve vaccine efficacy. Several clinical trials are currently underway to assess the efficacy of mRNA vaccines in combination with other oncology treatments. These clinical trials will help to identify safer and more effective antitumor therapies to improve the survival of cancer patients.

In summary, mRNA cancer vaccines constitute a potent and versatile form of immunotherapy. With increasing numbers of clinical studies, especially with regard to personalized vaccines, there is a growing possibility of developing mRNA vaccines against different cancers. Despite the promise of mRNA therapy, a great deal of research remains to be done. Future research should involve technology research and address clinical development. For technology research, researchers should focus on developing stable mRNA without impurities and safe advanced delivery systems. With regard to clinical research, further clinical trials for different tumors are required. In addition, the further development of personalized vaccines is required to improve patient survival and quality of life.

## Author Contributions

ZD and YT wrote the manuscript and constructed the figures. PY and JS revised the manuscript and figures. GA revised the grammatical and syntax errors throughout the manuscript. All authors contributed to the article and approved the submitted version.

## Conflict of Interest

The authors declare that the research was conducted in the absence of any commercial or financial relationships that could be construed as a potential conflict of interest.

## Publisher’s Note

All claims expressed in this article are solely those of the authors and do not necessarily represent those of their affiliated organizations, or those of the publisher, the editors and the reviewers. Any product that may be evaluated in this article, or claim that may be made by its manufacturer, is not guaranteed or endorsed by the publisher.
